# Synthesis of a Triazaisotruxene-Based Porous Organic Polymer and Its Application in Iodine Capture

**DOI:** 10.3390/molecules27248722

**Published:** 2022-12-09

**Authors:** Rong Gao, Bohang An, Cen Zhou, Xiao Zhang

**Affiliations:** 1 Fujian Key Laboratory of Polymer Materials, Fujian Provincial Key Laboratory of Advanced Materials Oriented Chemical Engineering, College of Chemistry and Materials Science, Fujian Normal University, Fuzhou 350007, China; 2 Fujian Engineering and Research Center of New Chinese Lacquer Materials, College of Materials and Chemical Engineering, Minjiang University, Fuzhou 350108, China

**Keywords:** porous organic polymers (POPs), triazaisotruxene, triazatruxene, crosslinking, iodine capture

## Abstract

A new triazaisotruxene-based porous organic polymer (POP) was designed and successfully synthesized by a FeCl_3_-promoted crosslinking reaction. As a result of its porosity and good thermal stability, the designed POP can be utilized as a promising adsorbent for iodine, not only in the gaseous phase, but also in organic and aqueous solutions. Compared to its triazatruxene (TN) analogue, the ITN-based POP shows equal iodine uptake in the gaseous phase and in hexane solution, and better uptake in aqueous solution.

## 1. Introduction

Porous organic polymers (POPs), which are composed of lightweight elements linked by strong covalent bonds, have become a research hotspot in recent years [1,2,3,4,5,6,7,8]. As a result of their large specific surface area, tunable pore sizes, and good thermal and chemical stability, POPs are extensively applied in various fields, such as gas storage and separation [9,10,11,12], pollutant removal [13,14,15,16,17], organic electronics [18,19,20], heterogeneous catalysis [21,22,23,24,25], etc. In order to endow them with new functionalities, the design and synthesis of POPs bearing novel structural motifs, especially polycyclic aromatic cores, is highly desired.

Among various polycyclic aromatic molecules, triazatruxene (TN) is a fully aromatic compound with a C_3h_ symmetrical structure, which can be regarded as an indole cyclo-trimer [26,27]. TN and its derivatives have been frequently used in the fields of liquid crystals [28], non-linear optics [29], organic light-emitting diodes (OLEDs) [30,31], organic field-effect transistors (OFETs) [32], organic photovoltaic (OPV) cells [33,34], etc. Moreover, TN has also been applied as a building unit for cage molecules and porous materials [35,36,37,38]. As an isomer of TN, triazaisotruxene (ITN) bears a triindole skeleton but features an asymmetrical structure [39]. Specifically, two NH groups are adjacent to each other, which result in chelating effects. Compared with the broad research interest in TN, little attention has been paid to the properties and applications of ITN, although it was first synthesized at nearly the same time as TN by Bergman et al. about 40 years ago [40]. So far, research on ITN is essentially an unexplored area.

To overcome the energy shortages and environmental concerns originating from the burning of fossil fuels, the use of reliable nuclear power has been growing. However, radioactive substances emitted from nuclear plants are severely hazardous to the natural environment and to human health. ^129^I and ^131^I formed by the fission of uranium atoms are the two main ingredients of nuclear waste, especially ^129^I, which has an ultra-long radioactive half-life (t_1/2_ = 1.57 × 10^7^ years) [41]. Thus, the search for appropriate adsorbents to capture iodine with long-term storage is crucial. Although various adsorbents such as silver zeolites [42,43], activated carbons [44,45], mesoporous silica [46,47], and metal organic frameworks (MOFs) [48,49] have been employed for iodine capture, POPs are still attractive candidates in this field because of their inherent properties [50,51].

Herein, we report the synthesis of a new ITN-based porous organic polymer through a FeCl_3_-promoted crosslinking reaction. After structural characterization, a morphological study, and an assessment of its thermal stability, ITNs potential in iodine capture is fully investigated. In the meantime, a porous organic polymer with a TN core is synthesized for comparison. Overall, the ITN-based porous organic polymer shows better or equal iodine capture ability in various tests.

## 2. Results and Discussion

### 2.1. Synthesis and Characterization of ITN-POP and TN-POP

**Scheme 1:**
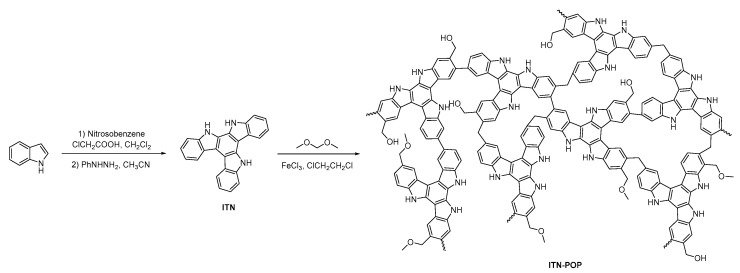

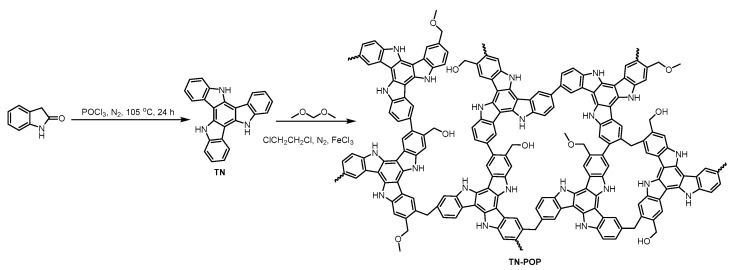
Synthesis of ITN-POP and TN-POP.

**Figure 1:**
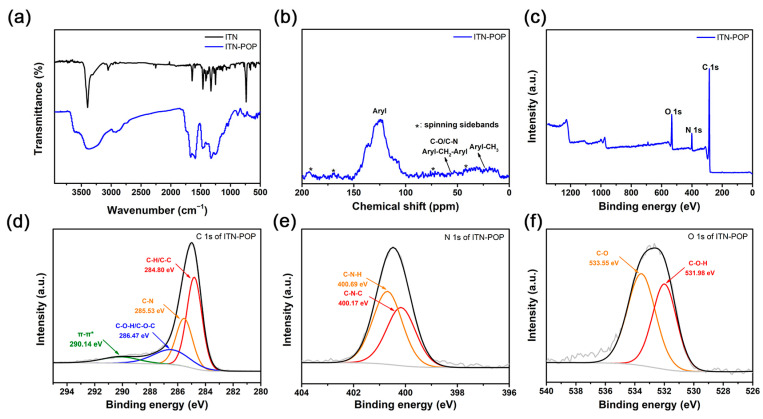
(**a**) FT-IR spectrum of ITN and ITN-POP; (**b**) Solid-state ^13^C CP/MAS NMR spectrum of ITN-POP; (**c**) XPS survey scan of ITN-POP; (**d**) C 1s of ITN-POP; (**e**) N 1s of ITN-POP; (**f**) O 1s of ITN-POP.

The synthetic routes for ITN-POP are shown in Scheme 1. An ITN monomer was prepared according to a procedure reported in [52]; then, it was reacted with formaldehyde dimethyl acetal (FDA) to obtain a crosslinking polymer. FT-IR analysis and ^13^C cross-polarization magic-angle spinning (CP/MAS) NMR measurements verified the successful synthesis of ITN-POP. In the FT-IR spectrum (Figure 1a), the peak at approximately 3400 cm^−1^ corresponded to the N-H stretching mode. The signals at 1670 cm^−1^, 1590 cm^−1^, and 1461 cm^−1^ were attributed to the stretching vibration of the aromatic ring. Notably, the saturated C-H vibration signals at around 2900–3000 cm^−1^ were attributed to the methylene linkers, indicating that the polymer was highly crosslinked. The CP/MAS NMR spectrum showed signals at 105–145 ppm assigned to aromatic moieties. Moreover, the broad peaks in the upfield region could be attributed to the methylene carbons between aryl groups and C (sp^3^)-O carbons (Figure 1b). In order to further investigate the elementary composition and the bonding types of ITN-POP, elemental analysis and X-ray photoelectron spectroscopy (XPS) measurements were conducted, confirming the existence of C, N, and O elements (Table S1, Figure 1c). The deconvoluted C 1s core energy spectrum of ITN-POP showed peaks at 284.80 eV and 285.53 eV that were ascribed to C-H/C-C and C-N. Meanwhile, the high-resolution C 1s spectrum of ITN-POP displayed the binding energies at 286.47 eV and 290.14 eV, which could be assigned to C-O-C/C-O-H and π–π* excitation (Figure 1d). The deconvoluted N 1s and O 1s core energy spectrum showed two peaks that were assigned to C-N and C-O moieties, respectively (Figure 1e,f).

**Figure 2:**
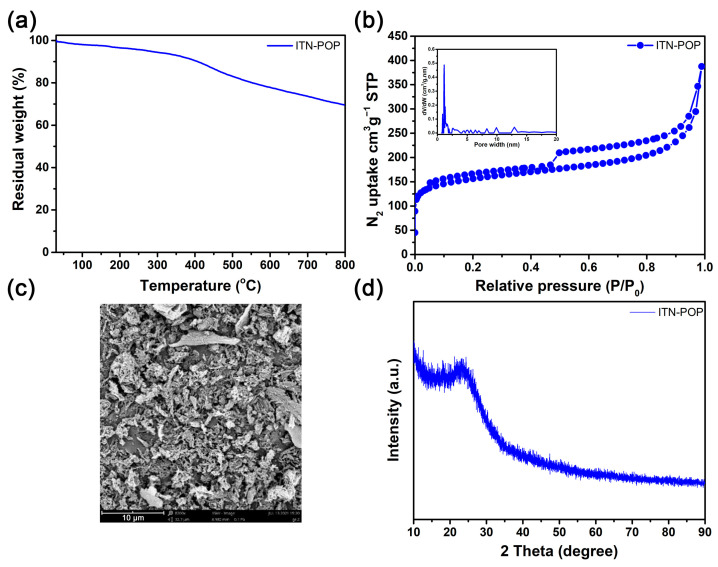
(**a**) TGA curve of ITN-POP; (**b**) N_2_-physisorption isotherm and pore size distribution curve (inset) of ITN-POP; (**c**) SEM image of ITN-POP; (**d**) PXRD spectrum of ITN-POP.

Thermogravimetric analysis (TGA) showed that ITN-POP was stable up to 406 °C (10% weight loss, Figure 2a). The N_2_ adsorption and desorption isotherms of ITN-POP at 77K showed a steep nitrogen gas uptake at a low relative pressure (P/P_0_ < 0.1) with hysteresis loops, indicating the presence of a high percentage of micropores and a minor proportion of mesopores (Figure 2b). The Brunauer–Emmett–Teller (BET) surface area of ITN-POP was measured to be 502.1 m^2^/g, with a total pore volume of 0.33 cm^3^/g. Scanning electron microscopy (SEM) images demonstrated that ITN-POP has an irregular morphology (Figure 2c). Its amorphous structure was further confirmed by its powder X-ray diffraction (PXRD) profile, which did not exhibit any assignable peaks (Figure 2d).

**Figure 3:**
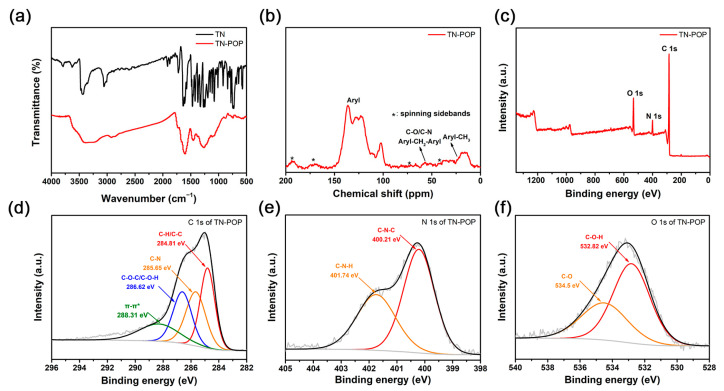
(**a**) FT-IR spectrum of TN and TN-POP; (**b**) Solid-state ^13^C CP/MAS NMR spectrum of TN-POP; (**c**) XPS survey scan of TN-POP; (**d**) C 1s of TN-POP; (**e**) N 1s of TN-POP; (**f**) O 1s of TN-POP.

**Figure 4:**
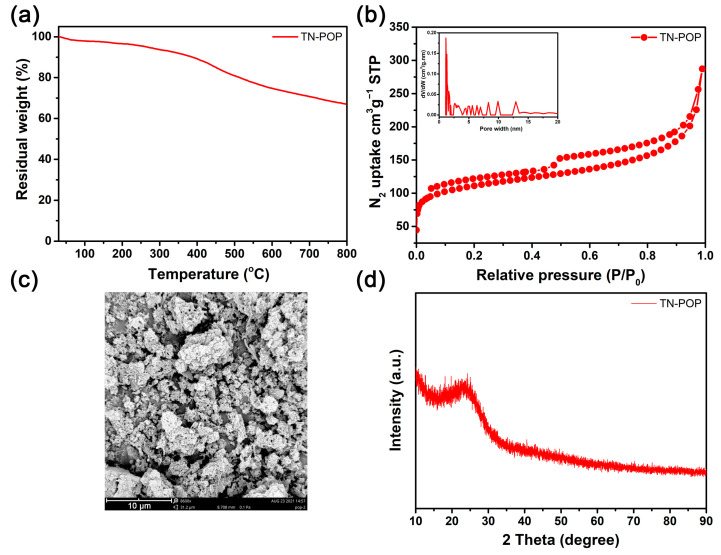
(**a**) TGA curve of TN-POP; (**b**) N_2_-physisorption isotherm and pore size distribution curve (inset) of TN-POP; (**c**) SEM image of TN-POP; (**d**) PXRD spectrum of TN-POP.

TN-POP was synthesized and characterized in the same manner as ITN-POP (Scheme 1 and Scheme S1, Figure 3 and Figure 4). In the FT-IR spectrum (Figure 3a), the peak at approximately 3400 cm^−1^ corresponded to the N-H stretching mode. The signals at around 1456 cm^−1^ and 1600 cm^−1^ were ascribed to the stretching vibration of the aromatic ring. Moreover, the saturated C-H vibration signals at around 2900–3000 cm^−1^ were attributed to methylene linkers and can also be found as evidence for successful crosslinking. In the CP/MAS NMR spectrum, the signals at 100–150 ppm can be attributed to aromatic carbons. The broad peaks in the upfield region could be attributed to the methylene carbons between aryl groups and C (sp^3^)-O carbons (Figure 3b). Elemental analysis and X-ray photoelectron spectroscopy (XPS) measurements demonstrated that TN-POP consists of C, N, and O elements (Table S1, Figure 3c–f). Thermogravimetric analysis (TGA) showed that TN-POP was stable up to 387 °C (10% weight loss, Figure 4a). From the N_2_ adsorption and desorption isotherms of TN-POP (Figure 4b), the BET surface areas and total pore volumes were calculated to be 354.5 m^2^/g and 0.41 cm^3^/g, respectively. Scanning electron microscopy (SEM) images revealed that TN-POP has an irregular morphology (Figure 4c). In addition, its amorphous structure was further proven by its powder X-ray diffraction (PXRD) profile (Figure 4d).

### 2.2. Iodine Capture of ITN-POP and TN-POP

**Figure 5:**
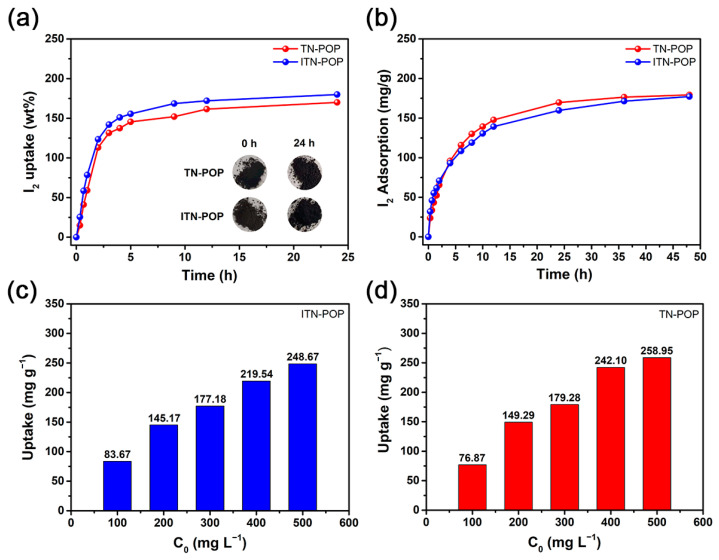
(**a**) Gravimetric uptake of iodine as a function of time at 348.15 K. Inserted photographs show the color change when ITN-POP and TN-POP were exposed to iodine vapor for 24 h; (**b**) Iodine adsorption by ITN-POP and TN-POP in cyclohexane solutions (300 mg L^−1^); (**c**,**d**) Kinetic studies of iodine adsorption by ITN-POP (**c**) and TN-POP (**d**) in cyclohexane solutions with different concentrations (100–500 mg L^−1^).

Considering their porous character and remarkable stability, ITN-POP and TN-POP could be promising absorbents for iodine. Thus, the iodine vapor-trapping capacities of these porous polymers were evaluated by gravimetric measurements. First, 20 mg of ITN-POP and TN-POP samples were placed in separate pre-weighed glass vials and exposed to a sealed container with excess iodine at 75 °C under an ordinary atmosphere, which are representative reprocessing conditions. As the iodine was captured, the color of the samples gradually changed from brown to black. The iodine-loaded samples were assessed by gravimetric measurements at selected time intervals, the results indicated that the iodine uptake of ITN-POP and TN-POP increased rapidly in the first 5 h, and the adsorption equilibrium was reached after 24 h. The equilibrium iodine uptakes for ITN-POP and TN-POP were measured to be 180 wt% and 170 wt%, respectively (Figure 5a), which are comparable to some metal–organic frameworks (MOFs) [53], nanoporous organic polymers (NOPs) [54], and conjugated microporous polymer nanotubes (CMPNs) [55].

In addition, the iodine adsorption capacities of ITN-POP and TN-POP in an organic solution were investigated. Five-milligram ITN-POP and TN-POP samples were added separately to an iodine–hexane solution with a concentration of 300 mg L^−1^ at room temperature. UV–Vis spectroscopy was used to evaluate the uptake and removal rate of iodine. The color of the iodine–hexane solutions gradually faded as the experiments went on (Figures S1 and S2). From the monitoring data, it was revealed that the adsorption rates of both POPs were relatively fast in the first 12 h, and then gradually slowed down until equilibrium (Figure 5b). For the kinetic study, the fractal-like pseudo-first-order (FL-PFO) model fitted the experimental data perfectly (R^2^ > 0.9990), revealing that the diffusion through micropores was the rate-controlling mechanism [56]. The equilibrium adsorption capacities for ITN-POP and TN-POP were evaluated to be 192.63 mg g^−1^ and 180.82 mg g^−1^, respectively (Figure S3). According to the standard curve (Figure S4), the removal rate was calculated to be about 60%. Moreover, the effect of different initial concentrations on iodine adsorption were also explored (Figure 5c,d). For both POPs, a higher iodine concentration in the solution resulted in a higher uptake value and a lower removal rate (Figure S5), and it was revealed once again that the iodine adsorption capacity of ITN-POP was similar to that of TN-POP.

**Figure 6:**
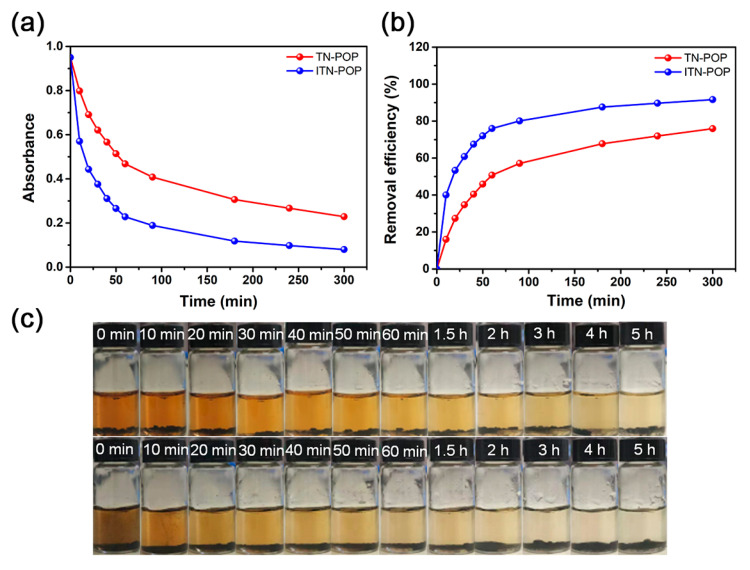
(**a**) The adsorption drops at 461 nm for ITN-POP and TN-POP containing iodine aqueous solutions; (**b**) Time-dependent adsorption profiles for ITN-POP and TN-POP in iodine saturated aqueous solutions (1.14 mM); (**c**) Photographs showing the color change for iodine aqueous solutions containing TN-POP (above) and ITN-POP (below).

Furthermore, the iodine adsorption capacities of ITN-POP and TN-POP in iodine-saturated aqueous solution (1.14 mM) were also investigated. First, 10 mg ITN-POP and TN-POP samples were immersed separately in 5 mL of saturated aqueous iodine solution, and the adsorption process was monitored by UV–Vis spectroscopy (Figure 6a,b Figure S7). During the experiment, an apparent color change in the aqueous iodine solution was observed from brown to nearly colorless. The absorbance decreased quickly during the first 1 h, and then gradually slowed down to 5 h (Figure 6c). The iodine removal rates for ITN-POP and TN-POP were 91.58% and 75.92%, respectively. The better uptake of ITN-POP might be attributed to its NH alignment and the resulting chelating effects, through which the porous material can extract iodine from a low-concentration solution more efficiently.

## 3. Materials and Methods

### 3.1. General Information

All reagents and solvents were purchased from Energy Chemical Co., Ltd. (Shanghai, China) or Sinopharm Chemical Reagent Co., Ltd. (Shanghai, China). ^1^H NMR spectra were recorded on a Bruker instrument (400 MHz) and internally referenced to a tetramethylsilane signal. Fourier-transform infrared spectra were recorded with a Nicolet Is50 FT-IR spectrophotometer. Solid-state ^13^C cross-polarization magic-angle spinning (CP/MAS) nuclear magnetic resonance measurements were performed on a Bruker AVANCE 400 WB MHz NMR system. X-ray photoelectron spectroscopy (XPS) measurements were performed on an ESCALAB 250 X-ray photoelectron spectroscope, using Al-Kα X-ray as the excitation source. Thermogravimetric analysis (TGA) profiles were recorded on a METTLER TGA/SDTA 851 thermal analyzer. The nitrogen adsorption–desorption isotherms at 77 K were measured using Micromeritics ASAP2460 analyzers, and the BET surface area was estimated by Brunauer–Emmett–Teller (BET) theory. Scanning electron microscopy (SEM) images were recorded using a Phenomenon LE electron microscope. Powder X-ray diffraction (PXRD) was recorded on a PANalytical X’pert PRO X-ray Diffractometer using Cu-Kα radiation in the 2*θ* range of 10–90°. Elemental analysis was calculated using Elementar Vario EL Cube. UV–Vis absorption spectra were recorded using a SHIMADZU UV-1750 spectrophotometer.

### 3.2. Synthesis of ITN and ITN-POP

Synthesis of triazaisotruxene (ITN)**:** Nitrosobenzene (11.0 g, 0.103 mol) was dissolved in 40 mL dichloromethane (DCM) and added dropwise under stirring to the mixture of indole (12.0 g, 0.102 mol) and chloroacetic acid (9.73 g, 0.103 mol) at room temperature. The reaction was kept for 12 h before working up. The resulting mixture was filtered and washed with DCM to obtain a green solid. Then, the green solid was dissolved in acetonitrile (30 mL), and phenylhydrazine (1.33 g, 12.3 mmol) was added. The mixture was stirred at room temperature for 3 h. The resulting precipitate was filtered, washed with DCM and dried under vacuum, affording ITN as a gray solid (2.42 g, 21% in 2 steps). ^1^H NMR (400 MHz, DMSO-*d_6_*): δ 11.88 (s, 1H), 11.49 (s, 1H), 11.39 (s, 1H), 8.88–8.79 (m, 3H), 7.83 (m, 3H), 7.51–7.38 (m, 6H).

Synthesis of ITN-POP: Following a reported procedure in the literature [57], ITN (500 mg, 1.45 mmol) was dissolved in anhydrous 1,2-dichloroethane (15 mL), then formaldehyde dimethyl acetal (0.73 mL, 8.69 mmol) and iron (III) chloride (1.41 g, 8.69 mmol) were added. The mixture was stirred at room temperature for 1 h under nitrogen, and then heated at 90 °C for 47 h. After cooling down to room temperature, methanol (50 mL) was added. The resulting mixture was stirred for 1 h, and the precipitate was collected by filtration. After washing with methanol, the obtained solid was vigorously stirred in an aqueous HCl solution (37%) for 2 h. The suspension was then filtered and washed with water, methanol, ethanol, acetone, and dichloromethane, in that order. After extraction with methanol in a Soxhlet extractor for 24 h, and then with tetrahydrofuran for another 24 h, the desired ITN-POP was collected as a brown solid (0.49 g).

### 3.3. Iodine Adsorption Capacity Measurements

#### 3.3.1. Iodine Vapor Uptake Capacity

First, 20 mg of ITN-POP or TN-POP was loaded into a glass vial, which was previously weighed and located in a sealed container with excess solid iodine kept at the bottom. The container was kept at 348.15 K at ambient pressure. After certain time intervals, the vial was taken out and weighed, and then loaded back into vapor of iodine to continue adsorption. The weight percentage of captured iodine was calculated through the following equation:

α = (m_2_ − m_1_)/m_1_ × 100%

where α represents the adsorption capacity, and m_1_ and m_2_ are the mass of ITN-POP or TN-POP sample before and after iodine intake, respectively.

#### 3.3.2. Iodine Adsorption from Solution

To evaluate the adsorption of dissolved iodine in cyclohexane, 5 mg of ITN-POP or TN-POP was immersed in 5 mL of n-hexane solution (300 mg L^−1^) containing iodine for 48 h. The adsorption process of iodine was monitored by UV–Vis spectroscopy. Similarly, for the iodine-saturated aqueous solution, 10 mg of ITN-POP or TN-POP was immersed in the solution, and other operations were performed as previously described. The iodine removal efficiency (%) was calculated through the following equation:

Iodine removal efficiency (%) = (C_0_ − C_t_)/C_0_ × 100%

where C_0_ and C_t_ represent the concentration of iodine before and after adsorption, respectively, which are proportional to absorbance.

## 4. Conclusions

In summary, we have synthesized a new triazaisotruxene-based porous organic polymer (POP) and explored its potential for iodine capture. Compared to its triazatruxene (TN) analogue, the ITN-based POP shows equal iodine uptake in the gaseous phase and in hexane solution and better uptake in an aqueous solution. To the best of our knowledge, this is the first example of a triazaisotruxene-based porous material. The research of its applications in other fields, especially heterogeneous photocatalysis, is now ongoing in our laboratory.

## Data Availability

The data presented in this study are available on request from the corresponding authors.

## Supplementary Materials

The following supporting information can be downloaded at: https://www.mdpi.com/article/10.3390/molecules27248722/s1. Scheme S1: Synthesis of TN and TN-POP; Table S1: Elemental analysis of TN-POP and ITN-POP; Figure S1: UV-vis spectra of iodine n-hexane solution containing TN-POP and ITN-POP (5 mg) at various times; Figure S2: Photographs of iodine capture in iodine n-hexane solution; Figure S3: Non-linear plots for iodine capture process in iodine n-hexane solution by fractal-like pseudo-first-order (FL-PFO) model: q_t_ = q_e_ (1 − exp(−kt^α^)). P1, P2 and P3 represent the equilibrium adsorption capacity (q_e_, mg/g), the adsorption rate constant (k, h^−1^) and the heterogeneity parameter of the surface (α), respectively; Figure S4: (a) UV-Vis spectra of iodine n-hexane solution at different concentrations (left). (b) Standard curve plotted based on the absorbance at 521 nm (right); Figure S5: The adsorption removal rates of TN-POP (a) and ITN-POP (b) for iodine at different concentrations of n-hexane solution; Figure S6: (a) UV-Vis spectra of iodine aqueous solutions at different concentrations (left); (b) Standard curve plotted based on the absorbance at 461 nm (right). Figure S7: Time-dependent UV-Vis adsorption spectra of iodine aqueous solution recorded after contacting with TN-POP (a) and ITN-POP (b). References [57,58] are cited in the supplementary materials.

## Sample Availability

Samples of ITN, ITN-POP, TN and TN-POP are available from the authors.
