# Supplementary material for: Synthesis of a Triazaisotruxene-Based Porous Organic Polymer and Its Application in Iodine Capture
Source: Molecules. 2022 Dec 9;27(24):8722. doi: 10.3390/molecules27248722 (PMC9784556; doi:10.3390/molecules27248722)
Supplement: Supplementary file 1 [file molecules-27-08722-s001.zip › molecules-2059116-supplementary.pdf]

Supplementary Information for

Synthesis of a Triazaisotruxene-Based Porous Organic Polymer and its Application in Iodine Capture

Rong Gao[1], Bohang An[1], Cen Zhou[2,*] and Xiao Zhang[1,*]

1. Fujian Key Laboratory of Polymer Materials, Fujian Provincial Key Laboratory of Advanced Materials Oriented Chemical Engineering, College of Chemistry and Materials Science, Fujian Normal University, Fuzhou 350007, China
2. Fujian Engineering and Research Center of New Chinese Lacquer Materials, College of Materials and Chemical Engineering, Minjiang University, Fuzhou 350108, China

Table of Contents

1. General information 3
2. Synthesis of TN and TN-POP 4
3. Elemental analysis of TN-POP and ITN-POP 5

4 Iodine uptake experiments 5

5 References 8

6 Copies of NMR spectra 9

1. General information

Unless stated otherwise, all reactions were carried out in flame-dried glassware under a dry nitrogen atmosphere. All reagents were commercially purchased and used without any further purification. ^{1}H NMR spectra were recorded on a Bruker instrument (400 MHz) and internally referenced to tetramethylsilane signal. Data for ^{1}H NMR are recorded as follows: chemical shift (δ, ppm), multiplicity (s = singlet, d = doublet, t = triplet, m = multiplet or unresolved, br = broad singlet, coupling constant(s) in Hz, integration).

Powder X-ray diffraction (PXRD) was recorded on a PANalytical X'pert PRO X-ray Diffractometer using Cu-Kα radiation in the 2θ range of 10-90°. The nitrogen adsorption-desorption isotherms at 77 K were measured with Micromeritics ASAP2460 analyzers, and the BET surface area was estimated by the Brunauer-Emmett-Teller (BET) theory. The pore-size-distribution curves were obtained from the adsorption branches using non-local density functional theory (NLDFT) method. Fourier Transform Infrared spectra were recorded with a Nicolet Is50 FT-IR spectrophotometer. Scanning electron microscopy (SEM) images were recorded using a Phenomenon LE electron microscope. Thermogravimetric analysis (TGA) profiles were recorded on a METTLER TGA/SDTA 851 thermal analyzer. UV/vis absorption spectra were recorded using a SHIMADZU UV-1750 spectrophotometer. X-ray photoelectron spectroscopy (XPS) measurements were performed on an ESCALAB 250 X-ray photoelectron spectroscopy, using Al Kα X-ray as the excitation source. Solid state ^{13}C cross-polarization magic-angle spinning (CP/MAS) nuclear magnetic resonance measurements was performed on a Bruker AVANCE 400 WB MHz NMR system. Elemental analysis was calculated by Elementar Vario EL Cube.

2. Synthesis of TN and TN-POP

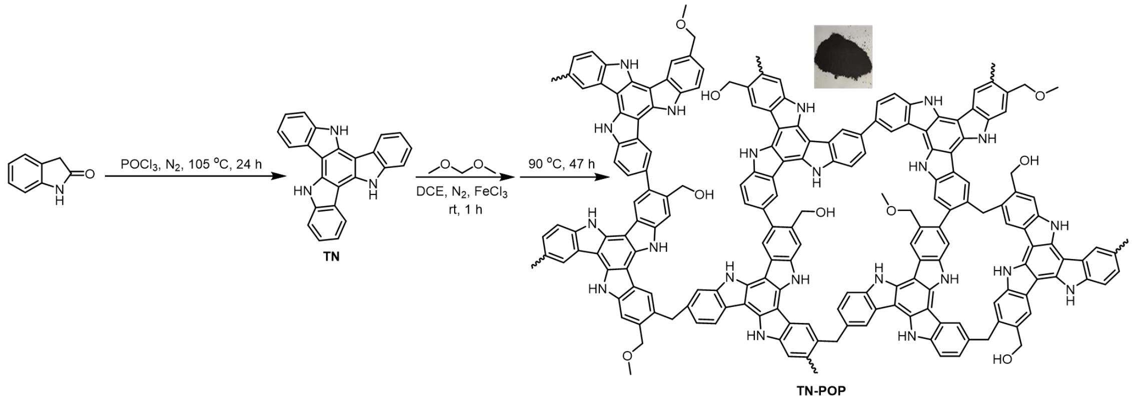

Scheme S1 Synthesis of TN and TN-POP

Synthesis of triazatruxene (TN): Following a literature reported procedure[1], A mixture of 2-indolinone (2.0 g, 0.015 mmol) and $POCl_3$ (8.4 mL) was heated at 105 °C for 24 h. Then, the reaction mixture was poured into ice and neutralized carefully with KOH. After neutralization, the precipitate was filtered and washed with plenty of water to give the crude product as a green solid. Further purification with column chromatography (PE/EA=8:1) give the desired product as a green powder (0.53 g, 10 %). ^1H NMR (400 MHz, DMSO-d_6): δ 8.84 – 8.75 (m, 3H), 7.79 (t, J = 8.4 Hz, 3H), 7.49 – 7.34 (m, 6H).

Synthesis of TN-POP: Following a literature reported procedure[2], **TN** (0.40 g, 1.2 mmol) was dissolved in anhydrous 1,2-dichloroethane (10 mL), then formaldehyde dimethyl acetal (0.59 mL, 6.95 mmol) and iron (III) chloride (1.13 g, 6.95 mmol) were added. The mixture was stirred at room temperature for 1 h under nitrogen, and then heated at 90 °C for 47 h. After cooled down to room temperature, methanol (50 mL) was added. The resulting mixture was stirred for 1 h and the precipitate was collected by filtration. After washing with methanol, the obtained solid was vigorously stirred in an aqueous HCl solution (37%) for 2 h. The suspension was then filtered and washed with water, methanol, ethanol, acetone and dichloromethane, subsequently. After extraction with methanol in a Soxhlet extractor

for 24 h, and then with tetrahydrofuran for another 24 h, The desired **TN-POP** was collected as a brown solid (0.42 g).

3. Elemental analysis of TN-POP and ITN-POP

Material	Exp. (%)		
	C	N	H
TN-POP	64.00	7.71	4.68
ITN-POP	63.57	8.32	4.65

Table S1 Elemental analysis of TN-POP and ITN-POP

4 Iodine uptake experiments

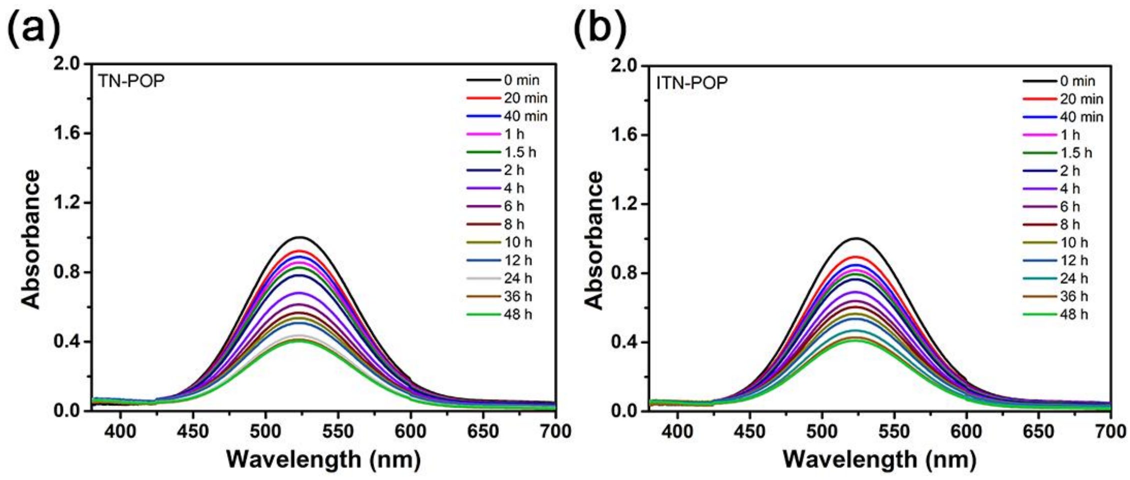

Figure S1 UV-vis spectra of iodine n-hexane solution containing TN-POP and ITN-POP (5 mg) at various times

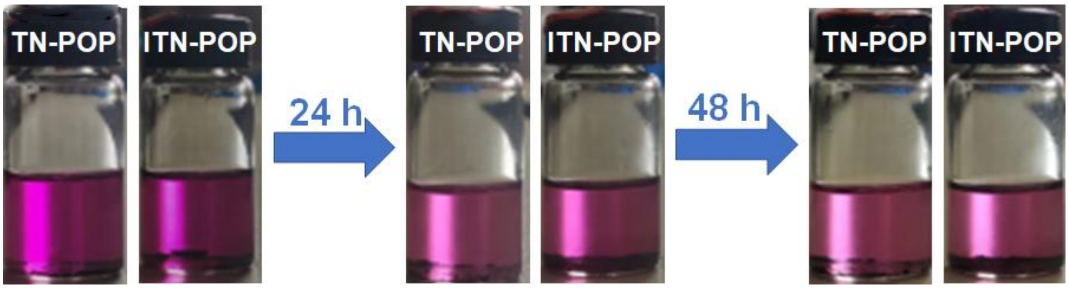

Figure S2 Photographs of iodine capture in iodine n-hexane solution.

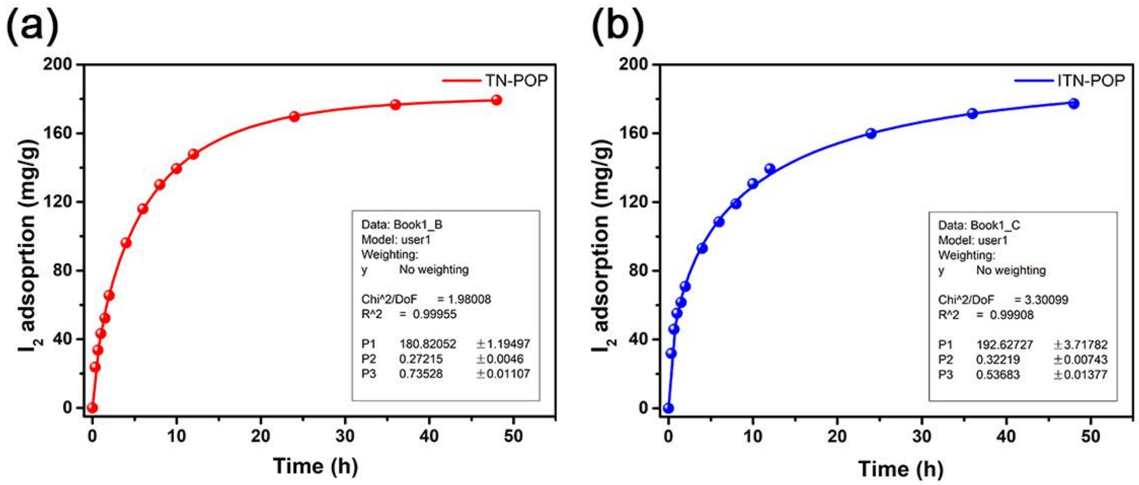

Figure S3 Non-linear plots for iodine capture process in iodine n-hexane solution by fractal-like pseudo-first-order (FL-PFO) model: $q_t = q_e (1 - \exp(-kt^{\alpha}))$. P1, P2 and P3 represent the equilibrium adsorption capacity (q_e, mg/g), the adsorption rate constant (k, h^{-1}) and the heterogeneity parameter of the surface (α), respectively.

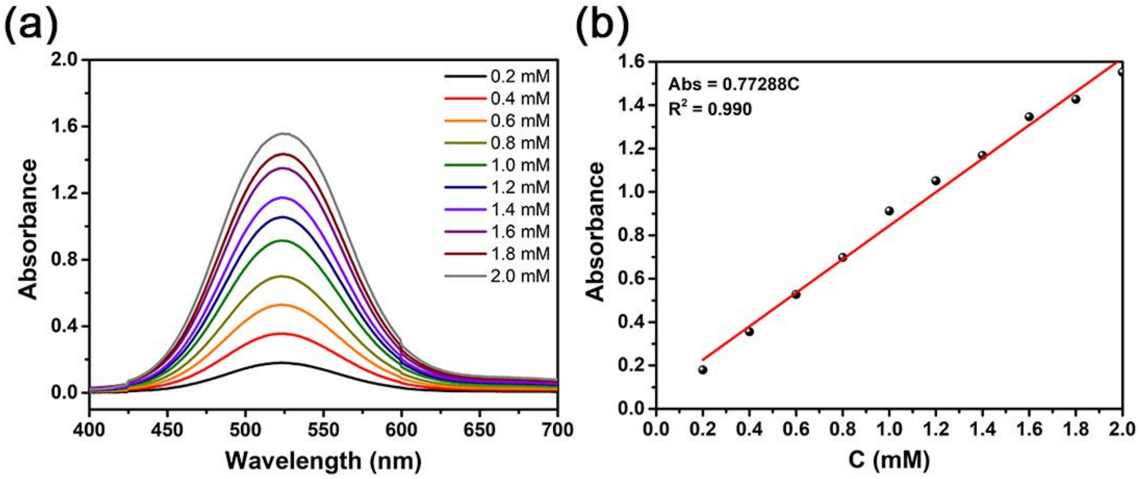

Figure S4 (a) UV-Vis spectra of iodine n-hexane solution at different concentrations (left). (b) Standard curve plotted based on the absorbance at 521 nm (right).

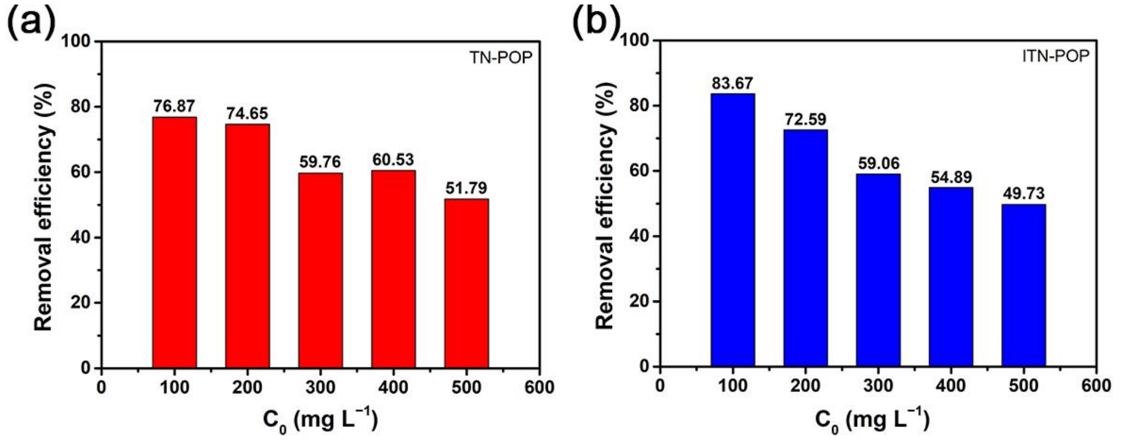

Figure S5 The adsorption removal rates of TN-POP (a) and ITN-POP (b) for iodine at different concentrations of n-hexane solution.

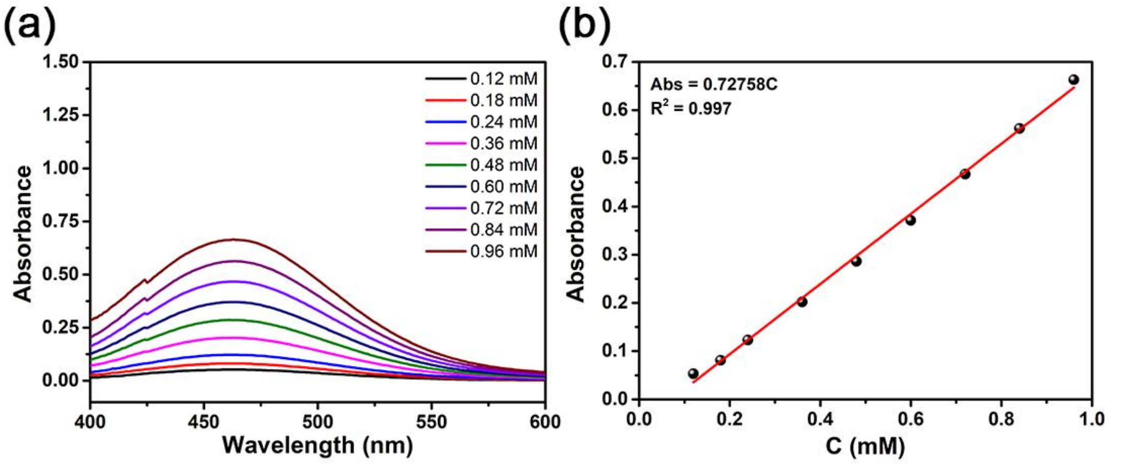

Figure S6 (a) UV-Vis spectra of iodine aqueous solutions at different concentrations (left); (b) Standard curve plotted based on the absorbance at 461 nm (right).

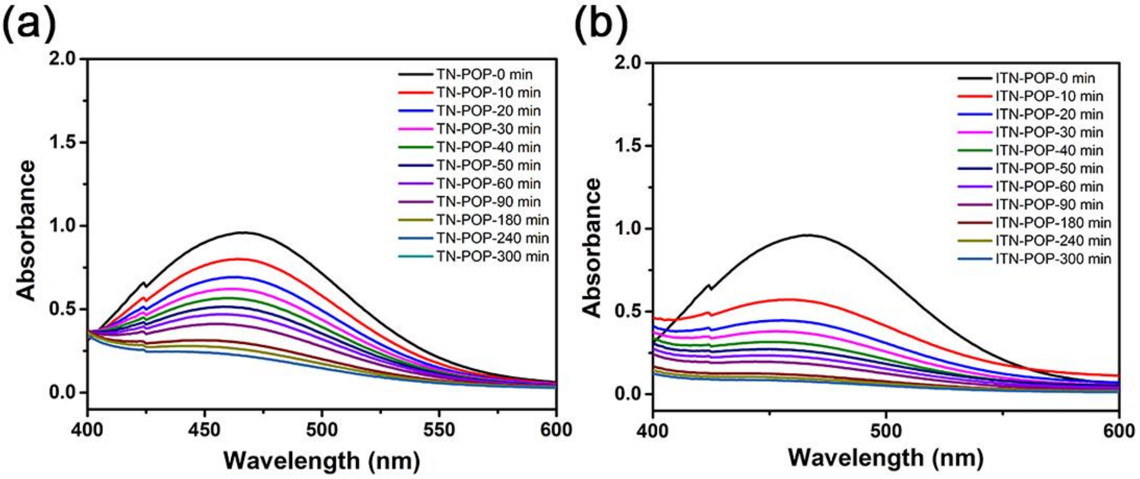

Figure S7 Time-dependent UV-Vis adsorption spectra of iodine aqueous solution recorded after contacting with TN-POP (a) and ITN-POP (b).

5 References

1. Rakstys, K.; Abate, A.; Dar, M. I.; Gao, P.; Jankauskas, V.; Jacopin, G.; Kamarauskas, E.; Kazim, S.; Ahmad, S.; Grätzel, M.; Nazeeruddin, M. K. *J. Am. Chem. Soc.* **2015**, 137, 16172-16178.
2. Zhu, J. -H.; Chen, Q.; Sui, Z. -Y.; Pan, L.; Yu, J. -H.; Han, B. -H. *J. Mater. Chem. A*. **2014**, 2, 16181-16189.

6 Copies of NMR spectra

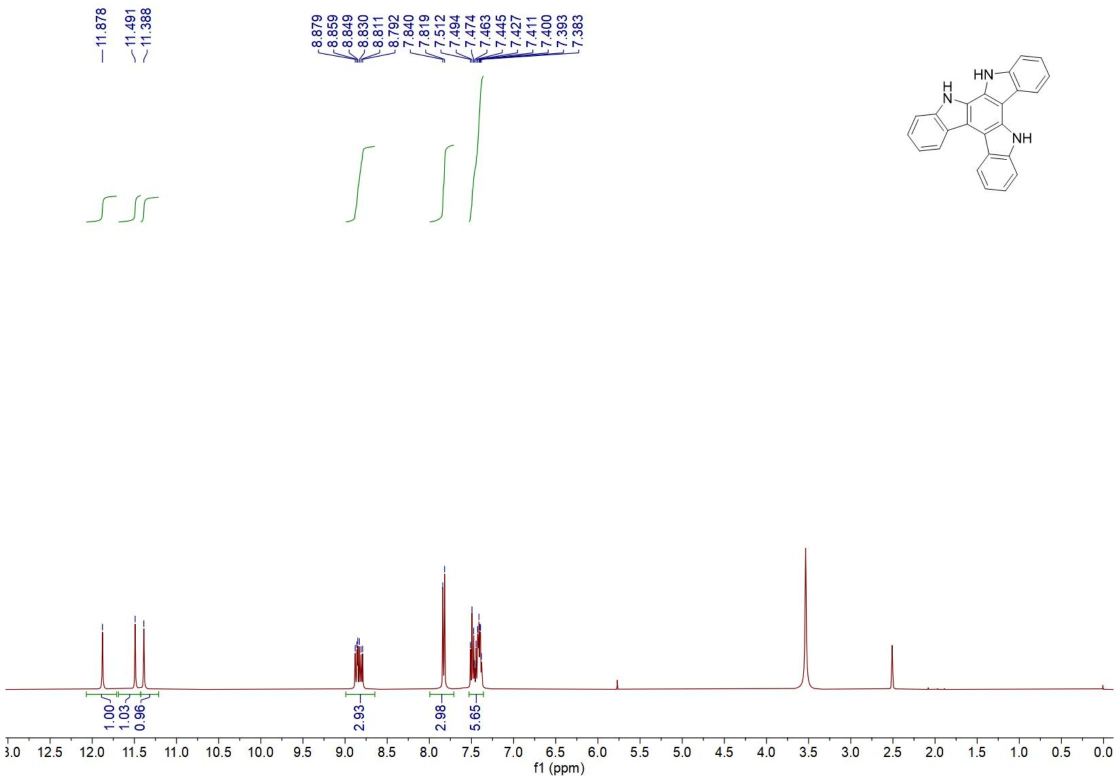

^{1}H NMR Spectrum of **ITN**

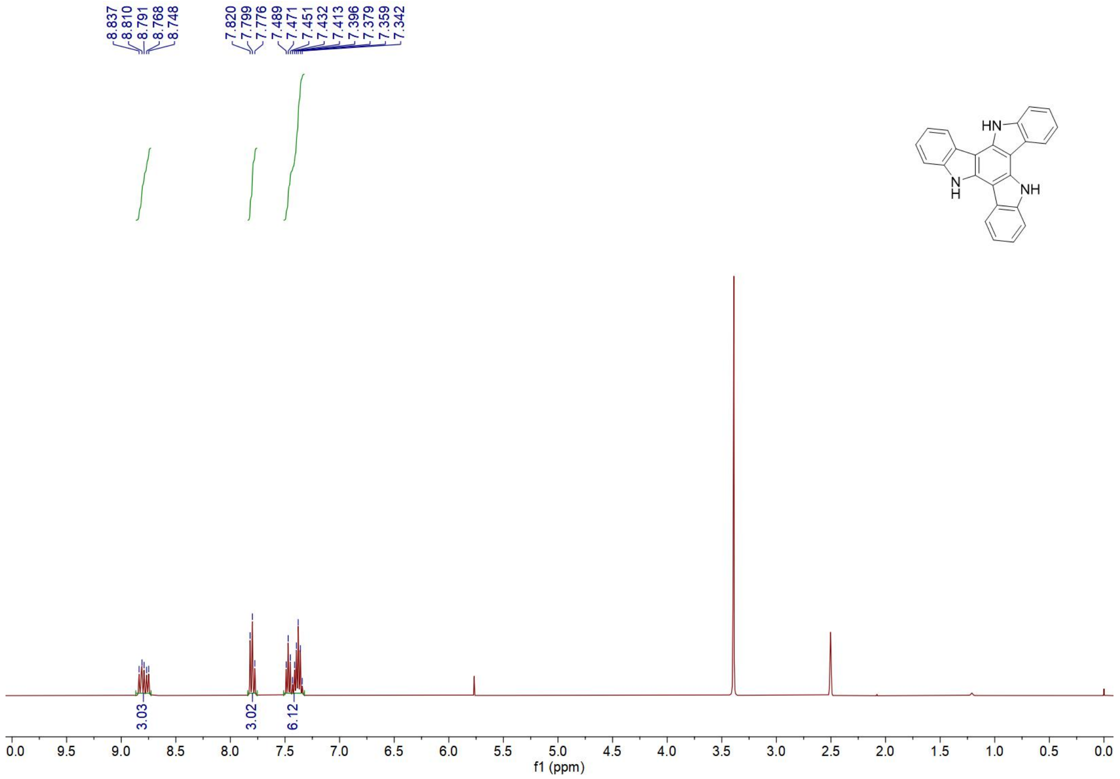

^{1}H NMR Spectrum of **TN**
